# Tousled-like kinase 1 is a negative regulator of core transcription factors in murine embryonic stem cells

**DOI:** 10.1038/s41598-017-18628-9

**Published:** 2018-01-10

**Authors:** Jina Lee, Min Seong Kim, Su Hyung Park, Yeun Kyu Jang

**Affiliations:** 1 Department of Systems Biology, College of Life Science and Biotechnology, Yonsei University, Seoul, 03722 Republic of Korea; 2 Initiative for Biological Function and Systems, Yonsei University, Seoul, 03722 Republic of Korea; 3 Present Address: Center for Genomic Integrity, Institute for Basic Science, Ulsan National Institute of Science and Technology, UNIST-gil 50, Ulsan, 689-798 Republic of Korea

## Abstract

Although the differentiation of pluripotent cells in embryonic stem cells (ESCs) is often associated with protein kinase-mediated signaling pathways and Tousled-like kinase 1 (Tlk1) is required for development in several species, the role of Tlk1 in ESC function remains unclear. Here, we used mouse ESCs to study the function of Tlk1 in pluripotent cells. The knockdown (KD)-based *Tlk1*-deficient cells showed that Tlk1 is not essential for ESC self-renewal in an undifferentiated state. However, *Tlk1*-KD cells formed irregularly shaped embryoid bodies and induced resistance to differentiation cues, indicating their failure to differentiate into an embryoid body. Consistent with their failure to differentiate, *Tlk1*-KD cells failed to downregulate the expression of undifferentiated cell markers including Oct4, Nanog, and Sox2 during differentiation, suggesting a negative role of Tlk1. Interestingly, Tlk1 overexpression sufficiently downregulated the expression of core pluripotency factors possibly irrespective of its kinase activity, thereby leading to a partial loss of self-renewal ability even in an undifferentiated state. Moreover, Tlk1 overexpression caused severe growth defects and G_2_/M phase arrest as well as apoptosis. Collectively, our data suggest that Tlk1 negatively regulates the expression of pluripotency factors, thereby contributing to the scheduled differentiation of mouse ESCs.

## Introduction

Embryonic stem cells (ESCs) are derived from the inner cell mass (ICM) of blastocysts and possess self-renewal and pluripotency capabilities^1,2^. Self-renewal is the process by which a stem cell divides and generates at least one daughter stem cell harboring a similar developmental ability to the mother stem cells^3^, whereas pluripotency is defined as the potential to form all three germ layer cell types including the mesoderm, endoderm, and ectoderm^4^. ESC pluripotency is controlled by the modulation of pluripotency transcription factors (TFs), including the core pluripotency TFs Oct4, Sox2, and Nanog, TF associated proteins, and chromatin modifications^2,5–9^. For example, levels of the POU TF Oct4 (encoded by *Pou5f1*) must be tightly controlled by fine-tuning to sustain the ESC status. Further, the downregulation of Oct4 results in the loss of pluripotency and the unscheduled induction of differentiation into the trophectoderm lineage, whereas an increase in Oct4 expression induces differentiation into the mesoderm and endoderm lineages^10^. The sex-determining region Y (SRY)-related high mobility group (HMG) box protein Sox2 is expressed in the ICM, epiblast, extraembryonic ectoderm, and in germ cells^11^. To maintain the pluripotency of stem cells, Sox2 levels must be tightly regulated similarly to other core pluripotency factors. The ablation of *Sox2* in embryos causes a failure in the formation of intact ESCs and induces differentiation into the trophectoderm or extraembryonic endoderm^11^. Nanog is a homeodomain-containing TF that maintains the self-renewal of ESCs independently of leukemia inhibitory factor (LIF) and contributes to the determination of the fate between the epiblast and primitive endoderm in blastocysts^12–14^. The depletion of Nanog in the ICM induces the differentiation into the primitive endoderm in parallel with the failure to establish the epiblast^13^. Further, the downregulation of Nanog in mouse ESCs (mESCs) leads to the induction of a broad range of lineage markers for the trophectoderm, mesoderm, ectoderm, and neural crest cells^15^. As well, Oct4, Sox2, and Nanog cooperatively regulate their target genes required for maintaining pluripotency and self-renewal and occupy the promoters of developmental genes associated with lineage specification whose expression is silenced in undifferentiated ESCs^2,16,17^.

The Tousled-like kinases (Tlk) are serine/threonine kinases that are evolutionarily conserved in both animals and plants^18^. *Tousled*, which was originally identified in the plant *Arabidopsis thaliana*, encodes a protein kinase that plays a role in both flower and leaf development^19^. *TLK1* and *TLK2* are mammalian homologs of *Tousled* that encode serine/threonine kinases that exhibit maximal activity in the S phase^20^. However, DNA damage induces the transient and rapid inactivation of TLKs via checkpoint kinase (Chk1)-dependent phosphorylation^21,22^. In *Drosophila melanogaster* and *Caenorhabditis elegans*, TLK depletion results in developmental arrest due to failures in proper chromatin organization and appropriate transcriptional regulation during development^23,24^. Existing data suggest that Tlk1 plays an important role in the regulation of development, but its functions in mESCs have not yet been investigated.

In this study, we investigated the roles of Tlk1 in mESC pluripotency and differentiation using gain- and loss-of-function approaches. Our results demonstrate that *Tlk1*-knockdown (KD) ESCs remained undifferentiated in the presence of LIF. In addition, *Tlk1*-depleted ESCs exhibited delayed silencing of pluripotency-related genes and maintained an undifferentiated state with high alkaline phosphatase (AP) activity even after the induction of differentiation. Conversely, the overexpression of Tlk1 in ESCs sufficiently abrogated the convex morphology and reduced AP activity. Interestingly, the ectopic expression of Tlk1 negatively controlled the expression of core ESC TFs and induced growth defects, most likely due to the arresting of ESCs in the G_2_/M-phase. Taken together, our data suggest that Tlk1 acts as a negative regulator of core pluripotency factors in mESCs.

## Results

### Tlk1 deficiency does not affect mESCs in the undifferentiated state in the presence of LIF

**Figure 1:**
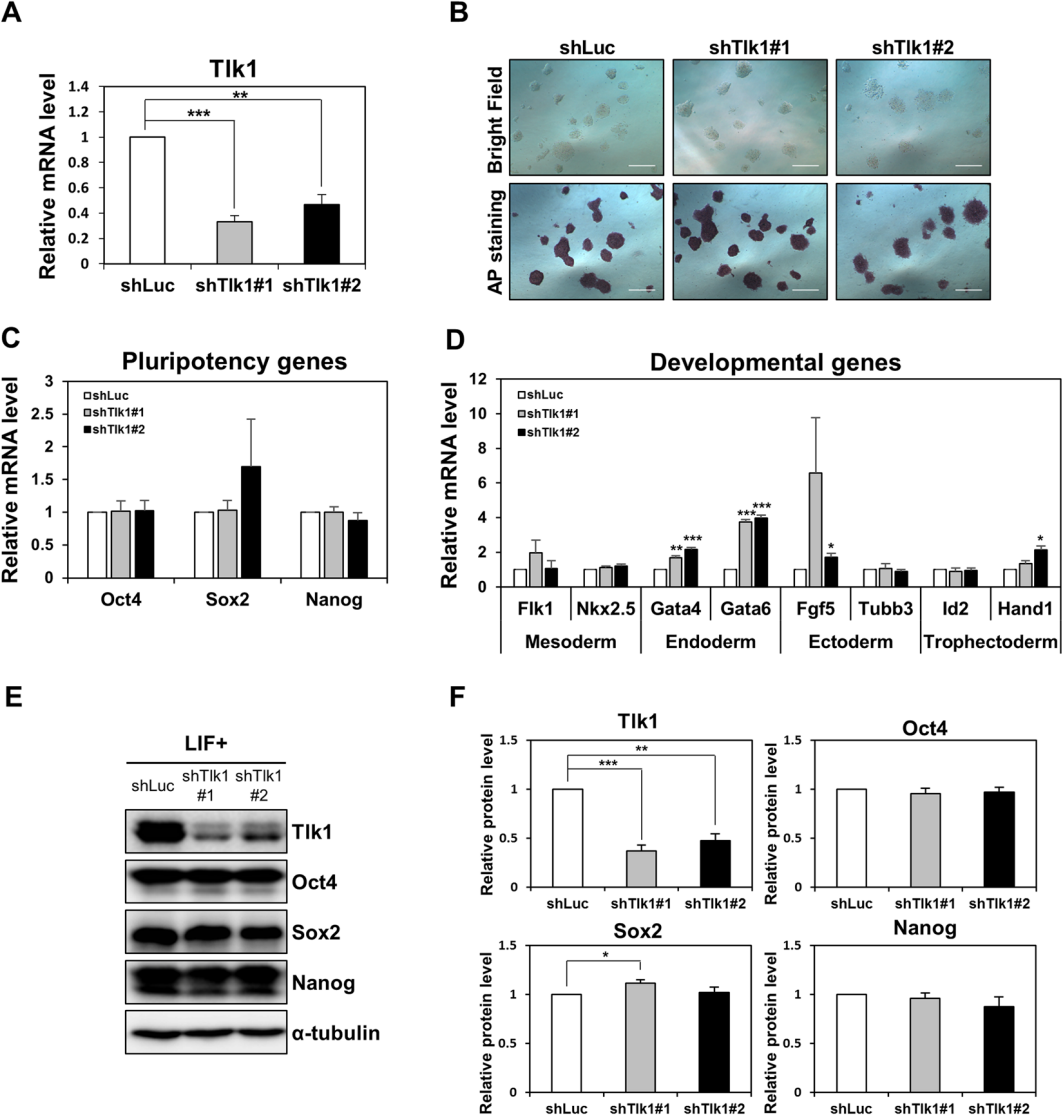
Tlk1 is not required for mESC self-renewal or pluripotency. (**A**) The efficiency of *Tlk1* knockdown (KD) in control (shLuc) and *Tlk1-*KD mESCs (shTlk1 #1 and #2) was confirmed by RT-qPCR analysis. Data are mean (n = 3) ± SEM. **Р < 0.01 and ***Р < 0.001. (**B**) The morphology of control (shLuc) and *Tlk1*-KD (shTlk1 #1 and #2) mESCs was evaluated using phase-contrast microscopic images and AP staining. Scale bars represent 500 µm. (**C** and **D**) The mRNA expression of pluripotency-associated and development-associated genes were analyzed by RT-qPCR in control (shLuc) and *Tlk1*-KD (shTlk1 #1 and #2) mESCs cultured under undifferentiated self-renewal conditions. All data were normalized to Gapdh and plotted relative to the expression level in control cells. Data are means (n = 3) ± SEM. *Р < 0.05, **Р < 0.01, and ***Р < 0.001. (**E**) The protein levels of pluripotency factors in control (shLuc) and *Tlk1*-KD (shTlk1 #1 and #2) mESCs was analyzed by immunoblotting using antibodies specific to Oct4, Sox2, and Nanog. (**F**) Quantification based on densitometry of Western blotting data from (**E**). All data were normalized to α-tubulin. Data are means (n = 3) ± SEM. *Р < 0.05, **Р < 0.01, and ***Р < 0.001.

To determine the role of Tlk1 in ESC function, we established *Tlk1*-KD mESCs using a shRNA-based RNAi method. We constructed two different shRNAs to avoid off-target effects and confirmed KD efficiency via qRT-PCR and Western blotting (Fig. 1A and E). The *Tlk1*-KD cells were morphologically indistinguishable from the control cells. In addition, no notable changes in AP staining were observed in the *Tlk1*-KD cells compared to the control KD cells (shLuc) under LIF-supplementation, suggesting that Tlk1 is not required for the self-renewal of ESCs (Fig. 1B). Next, we investigated the effects of *Tlk1* depletion on the expression of several genes involved in pluripotency or differentiation using qRT-PCR and found out that *Tlk1* deficiency did not affect the expression of pluripotency-associated genes, including *Oct4*, *Nanog*, and *Sox2* (Fig. 1C). Similarly, the expression of genes associated with early differentiation, namely *Flk1* and *Nkx2.5* for the mesoderm, *Fgf5* and *Tubb3* for the ectoderm, and *Id2* and *Hand1* for the trophectoderm, was not significantly changed in *Tlk1*-KD cells compared with control KD cells (Fig. 1D). However, the expression of other differentiation-associated genes (*GATA4* and *GATA6* for the endoderm) was moderately increased (Fig. 1D). Consistent with this mRNA expression profile, the Western blotting analysis revealed that the Oct4, Nanog, and Sox2 levels in *Tlk1* KD cells were not significantly changed relative to the control KD cells (Fig. 1E and F). Thus, these results suggest that, although it might not be necessary for mESC pluripotency and self-renewal, Tlk1 might regulate the expression of endoderm-associated genes.

### Tlk1 is required for the proper induction of scheduled differentiation

**Figure 2:**
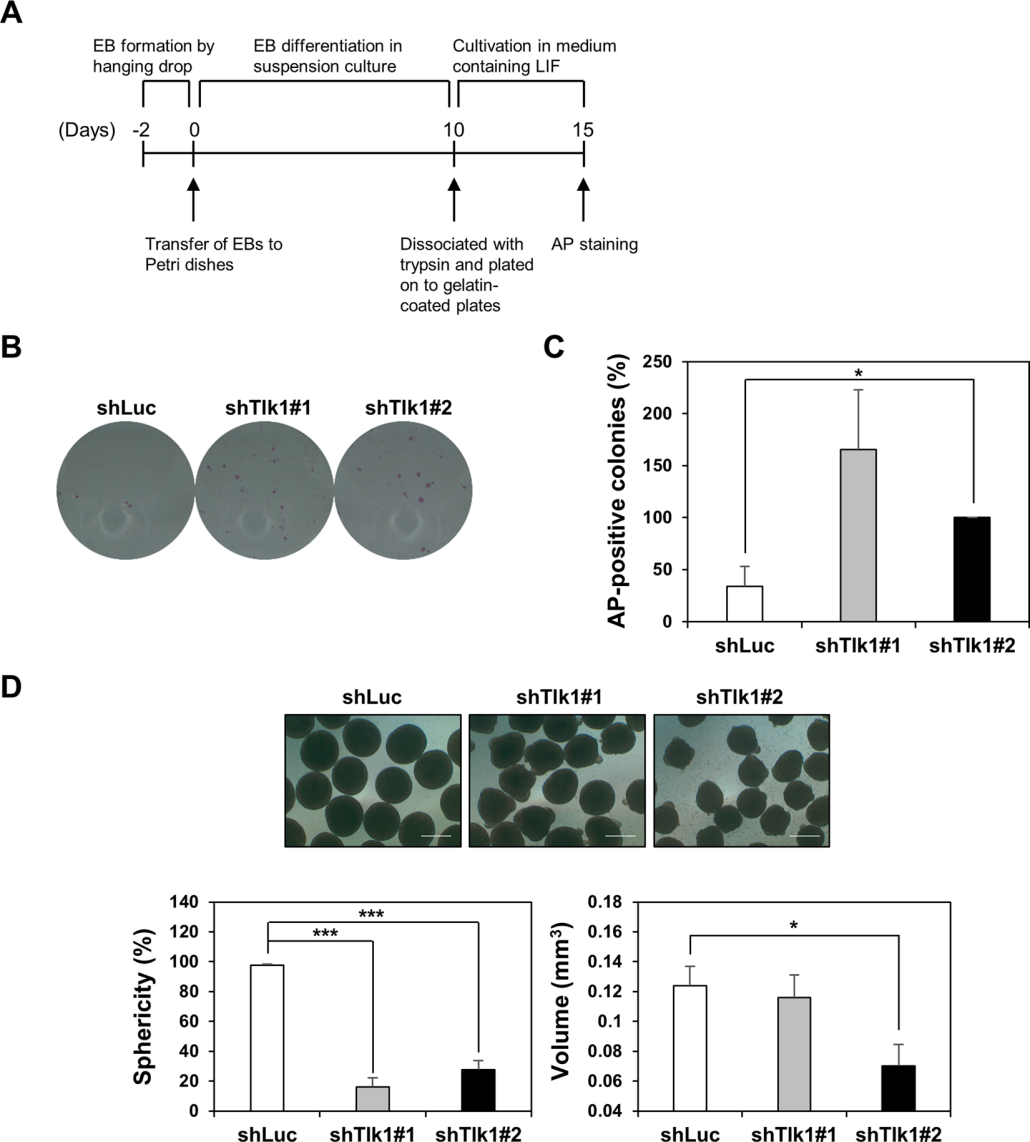
Depletion of Tlk1 impairs the scheduled differentiation of mESCs. (**A**) Schematic representation of commitment assay in control KD (shLuc) and *Tlk1*-KD (shTlk1 #1 and #2) mESCs. (**B**) The morphology of control (shLuc) and *Tlk1*-KD (shTlk1 #1 and #2) mESCs after EB formation was investigated by AP staining. (**C**) Quantification of results from (**B**). The areas of the AP positive colonies were measured using the Image J software. The areas of the AP-positive colonies (%) were normalized to that of shTlk1#2 cells ( = 100%). Data are means (n = 3) ± SEM. *Р < 0.05, **P < 0.01, and ***P < 0.001. (**D**) EB formation in control (shLuc) and *Tlk1*-KD (shTlk1 #1 and #2) mESCs was measured using a hanging drop assay. Scale bar, 500 µm. The quantification of EB sphericity and volume. Mean sphericity and volume analyzed using the AnaSP software and SEM of day 3 EBs from control (shLuc) and *Tlk1*-KD cells. Mean values (n = 4) ± SEM were plotted and 40 EBs were analyzed for each of the four independent experiments. Sphericity (%) is a proportion of EBs with a KD sphericity value above 0.9.

Because some differentiation-associated genes were aberrantly expressed in *Tlk1*-KD mESCs, we investigated whether the *Tlk1*-depleted cells were resistant to differentiation cues using a commitment assay, as previously described^25^. Embryoid bodies (EBs) can be used as a differentiation assay to test ESC pluripotency^25^. *Tlk1*-KD or control cells were allowed to form EBs for 12 days. The cells were then transferred to and cultured in media containing LIF for 5 days, and their differentiation patterns were assessed by AP staining (Fig. 2A). The EB-dependent differentiation of control KD cells (shLuc) proceeded normally without detectable delays in the presence of LIF, as confirmed by a low number of AP-stained colonies (Fig. 2B). Conversely, AP-positive ESC-like colonies were highly enriched in the EBs derived from two *Tlk1*-KD cell lines that were maintained in LIF-supplemented culture, indicating a failure of *Tlk1*-depleted mESCs to differentiate into an EB (Fig. 2B). Moreover, the failure to induce *Tlk1*-KD cells to differentiate was also supported by the quantitative analysis of AP-stained colonies (Fig. 2C). Because the delayed differentiation of *Tlk1*-KD cells might affect the formation of EBs, we examined if *Tlk1* depletion influenced EB formation and observed EB morphology using phase-contrast microscopy. We found that *Tlk1* depletion decreased the size of EBs and caused them to form irregular shapes (Fig. 2D). In addition, we randomly selected 40 EBs and measured their sphericity and volume. Our results revealed that *Tlk1* depletion significantly decreased the sphericity and volume of EBs, suggesting an impairment in the proper induction of differentiation into an EB (Fig. 2D, bottom panels).

**Figure 3:**
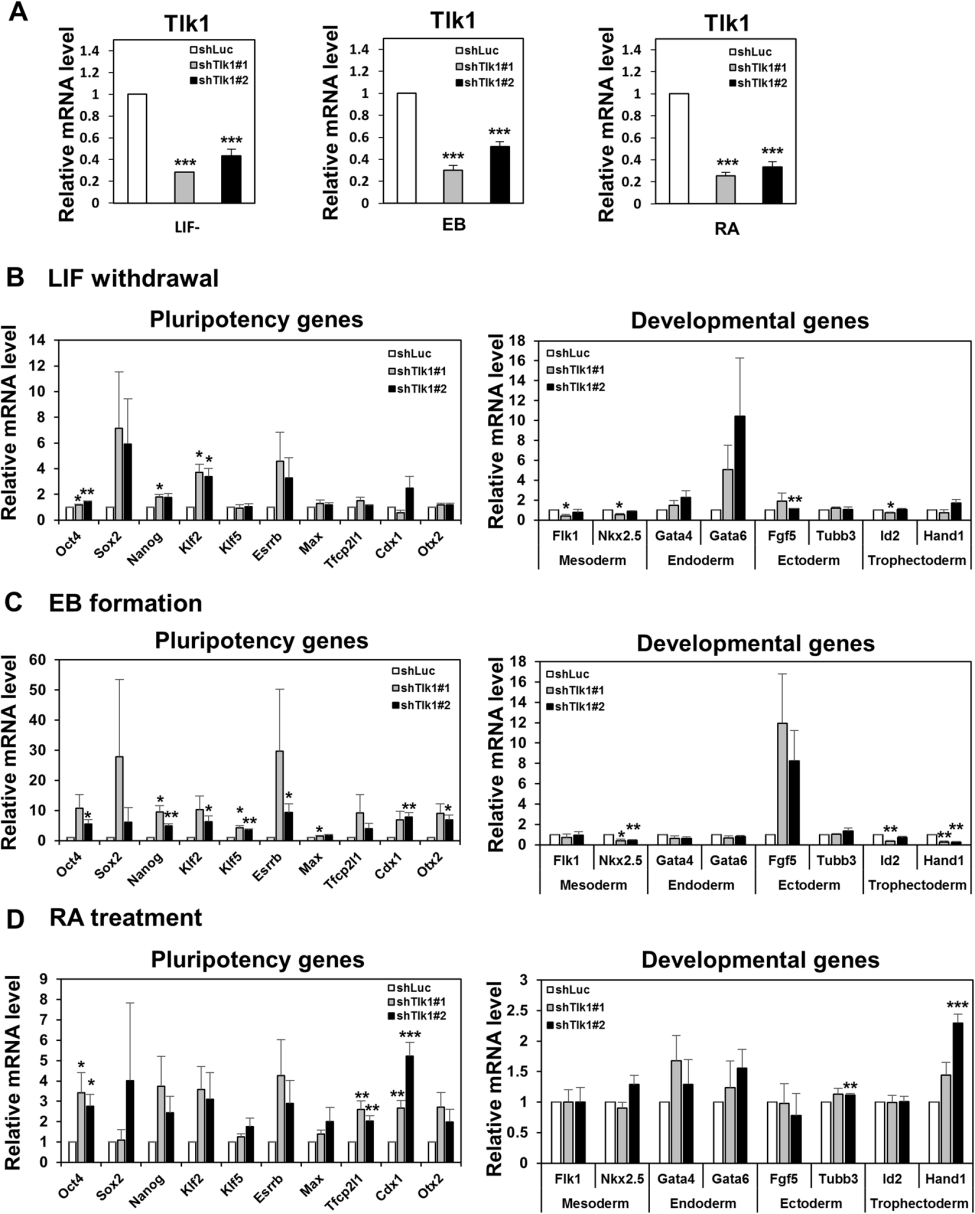
*Tlk1* deficiency leads to a failure in the scheduled downregulation of pluripotency-associated genes and the aberrant expression of lineage-associated genes. (**A**) The KD efficiency of *Tlk1* in control (shLuc) and *Tlk1*-KD (shTlk1 #1 and #2) mESCs cultured in three separate differentiation conditions was confirmed by RT-qPCR. Differentiation was induced by LIF withdrawal (LIF-), embryoid body formation (EB), and by retinoic acid treatment (RA). (**B–D**) The mRNA expression of pluripotency-associated and development-associated genes under three separate differentiation conditions including LIF withdrawal (**B**), embryoid body formation (**C**), and retinoic acid treatment (**D**) was analyzed by RT-qPCR. All data are normalized to Gapdh. The mRNA levels in luciferase (Luc)-KD control cells were normalized to 1. Data are means (n = 3) ± SEM for LIF- and EB and for RA (n = 4). *Р < 0.05, **Р < 0.01, and ***Р < 0.001.

**Figure 4:**
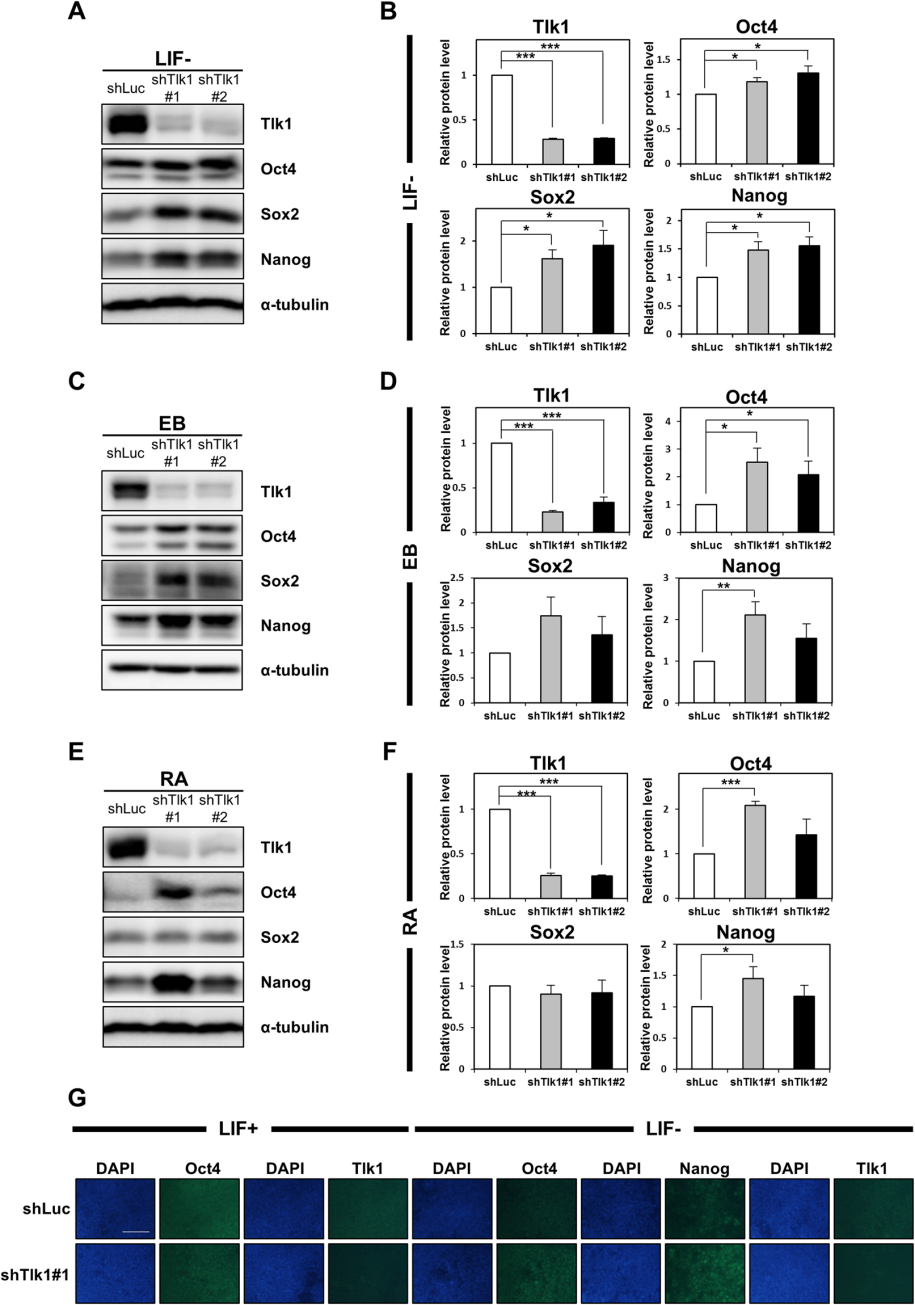
*Tlk1*-deficiency in mESCs causes a delay in the downregulation of core pluripotency factors upon differentiation. (**A**,**C** and **E**) Representative immunoblotting images showing Tlk1, Oct4, Sox2, and Nanog levels in *Tlk1*-KD cells upon differentiation. Differentiation was induced three different ways as previously described in Fig. 3. Alpha-tubulin was used as the loading control. (**B**,**D** and **F**) Quantification of the relative expression of the target proteins in panels (A,C, and E). The target proteins levels were normalized to that of α-tubulin. The protein expression levels of shLuc KD cells were normalized to 1. The biological data are presented as mean (n = 4) ± SEM for LIF- and EB and for RA (n = 3). *Р < 0.05, **P < 0.01, and ***P < 0.001. (**G**) Immunofluorescence analysis of Oct4, Nanog and Tlk1 in control (shLuc) and *Tlk1*-deficient mESCs. Scale bars represent 100 µm.

Because some differentiation-associated genes were upregulated by *Tlk1* depletion under LIF-supplemented conditions, we investigated whether *Tlk1* depletion affected gene expression in response to differentiation cues. The expression of pluripotency-associated or differentiation-associated genes under three separate differentiation-inducing conditions including LIF-withdrawal, EB formation, and retinoic acid (RA)-treatment was assessed using qRT-PCR. The KD efficiency in the *Tlk1*-depleted mESCs during differentiation was likewise confirmed by qRT-PCR (Fig. 3A). The proper induction of differentiation was also confirmed by the rapid downregulation of Oct4 in control KD cells (Supplementary Fig. S1). The differentiation-induced downregulation of pluripotency-related genes such as *Oct4*, *Sox2*, *Nanog*, *Klf2*, and *Esrrb* was delayed in *Tlk1-*KD cells relative to control KD cells (Fig. 3B,C and D). In contrast to pluripotency-associated gene expression, the induction of differentiation-associated genes was hindered during the differentiation of *Tlk1*-KD cells compared with control KD cells (Fig. 3B–D). In accord with the mRNA expression profiles, the Western blotting results confirmed that the Oct4, Nanog, and Sox2 levels were higher in differentiated *Tlk1*-KD ESCs compared with differentiated control KD cells (Fig. 4A–F). In addition, the immunostaining results revealed that Oct4 and Nanog levels were increased in *Tlk1*-KD cells compared with control cells (shLuc) during differentiation induced by LIF withdrawal (Fig. 4G). Thus, these results indicate that *Tlk1* depletion leads to the aberrant expression of differentiation-associated genes and the failure to downregulate the expression of pluripotency-associated factors during differentiation. Collectively, our findings suggest that Tlk1 is required for the proper induction of scheduled differentiation.

### Ectopic expression of Tlk1 is sufficient to induce the downregulation of core pluripotency factors

**Figure 5:**
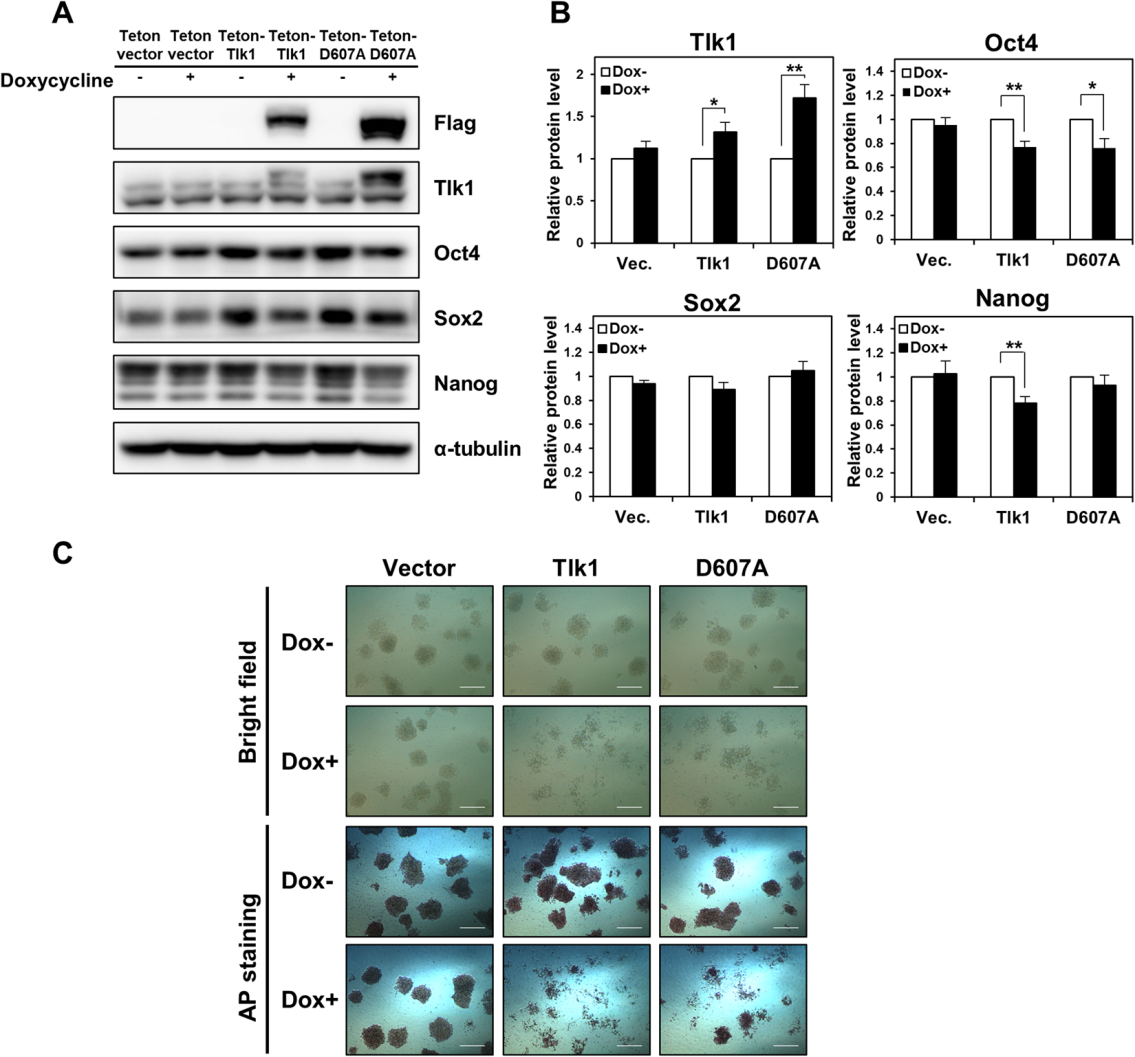
The forced expression of Tlk1 results in the aberrant downregulation of core pluripotency factors and attenuates self-renewal. (**A**) Immunoblot analysis of Oct4, Sox2, and Nanog levels in control mESCs (empty vector or doxycycline depletion) and Tlk1-overexpressing mESCs. The mESCs expressing an empty vector or the Tet-On-Tlk1 or Tet-On-Tlk1-D607A expression vector were cultured in the absence or presence of doxycycline (Dox; 100 ng/ml) for 24 hrs under undifferentiated self-renewal conditions. (**B**) Quantification of results from (**A**). The protein levels of the target genes were normalized to α-tubulin levels. The protein expression levels of each mESC line not treated with doxycycline were normalized to 1. The biological data are presented as mean (n = 6) ± SEM. *Р < 0.05, and **P < 0.01. (**C**) The morphology and AP staining of Tet-On-inducible Tlk1-expressing cell lines cultured in mock (Dox−) or doxycycline (Dox+) for 48 hrs. Scale bar, 500 µm.

Because *Tlk1* depletion caused the delayed differentiation of mESCs and we were unable to generate a mESC line that stably overexpressed Tlk1, which suggested that the overexpression of Tlk1 might cause lethality in mESCs, we investigated the effect of Tlk1 overexpression on mESC function. To test our hypothesis regarding the overexpression of Tlk1, we established mESCs that conditionally overexpressed Flag-tagged Tlk1 under the control of the Tet-On inducible expression system, which is a doxycycline-inducible promoter. We examined Oct4, Sox2, and Nanog levels by Western blotting, the results of which demonstrated that the steady-state levels of the core pluripotency factors were decreased following the overexpression of Flag-tagged wild-type Tlk1 (Fig. 5A and B). To determine if the kinase activity of Tlk1 was associated with the downregulation of the core pluripotency factors following the overexpression of Tlk1, we examined the effects of the overexpression of a D607A Tlk1 mutant. In humans, TLK1 harboring the D607A mutation is catalytically inactive and considered a kinase-dead mutant^20,26^. In this study, we mutated the Asp607 residue of Flag-tagged Tlk1 to alanine (D607A; kinase-dead mutant) because the Asp607 residue within the catalytic domain of Tlk1 is completely conserved between mice and humans. Our data revealed that the overexpression of the Flag-tagged Tlk1-D607A mutant also resulted in decreased levels of the core pluripotency factors, similar to wild-type Tlk1 (Fig. 5A and B). Moreover, Flag-tagged Tlk1-D607A mutant caused downregulation of the pluripotency factors in the condition of depletion of endogenous Tlk1 (Supplementary Fig. S4). Therefore, the results suggest that the overexpression of Tlk1 renders its kinase activity possibly unnecessary for the downregulation of the core pluripotency factors.

To assess the effects of Tlk1 overexpression on ESC self-renewal further, we examined the self-renewal capacity of mESCs overexpressing wild-type or D607A-mutant Tlk1 and found that the overexpression of Tlk1 led to morphological changes, including a diffuse epithelial appearance (Supplementary Fig. S2). Consistent with the apparent morphological changes, the AP staining of Tlk1-overexpressing cells was moderately reduced compared to control cells (Vector), even in the presence of LIF, which suggested a partial loss of self-renewal ability (Fig. 5C).

### Tlk1 overexpression causes growth defects and an increase in the G_2_/M phase population

**Figure 6:**
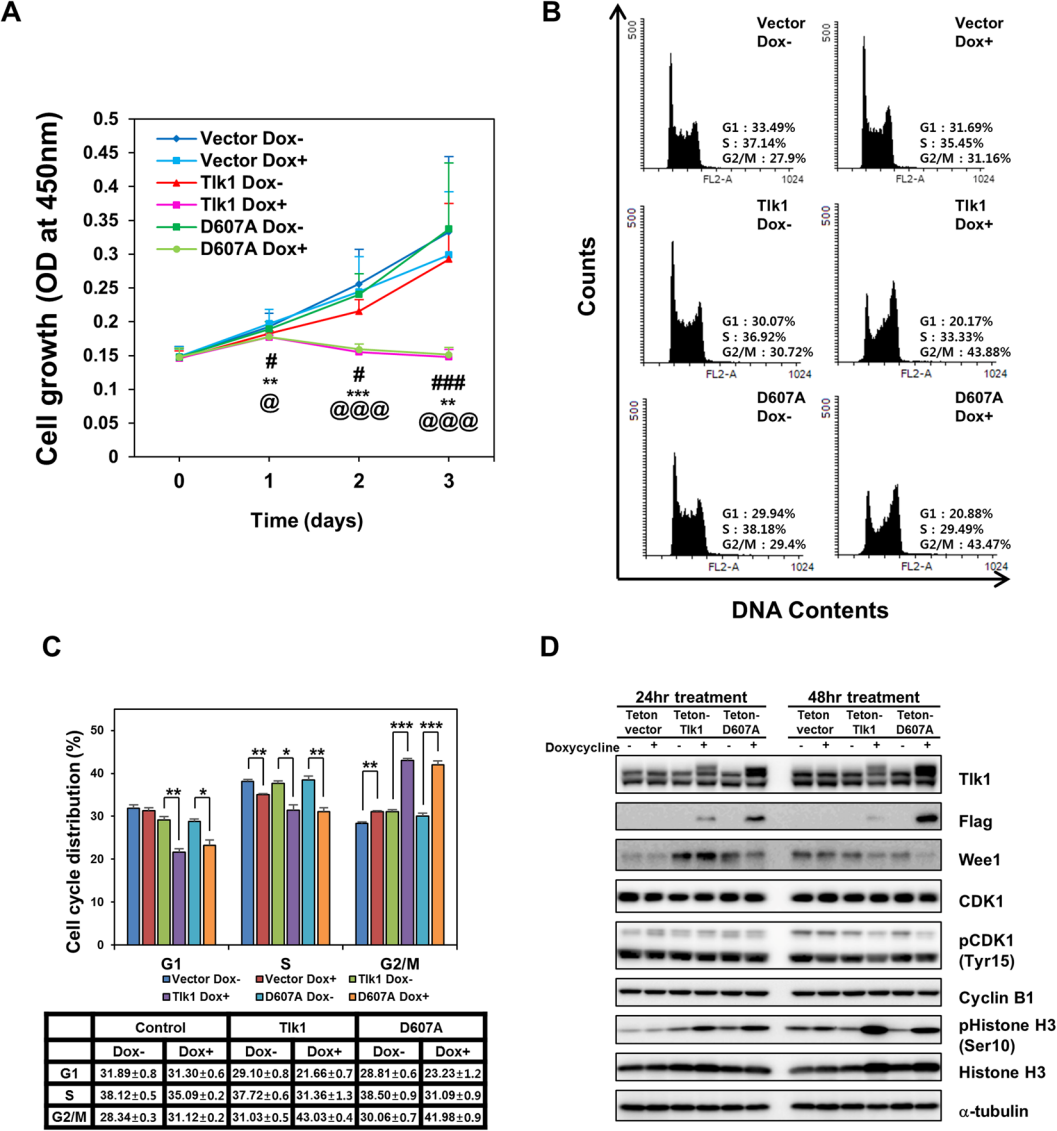
Overexpression of Tlk1 inhibits cell proliferation and induces the accumulation of G_2_/M phase cells in mESCs. (**A**) Ectopic expression of wild-type Flag-Tlk1 and Flag-Tlk1-D607A affects cell proliferation in mESCs. The overexpression of Flag-Tlk1 or Flag-Tlk1-D607A blocked cell growth compared to the control mESCs (empty vector or doxycycline depletion). The growth rate was measured using a CCK-8 assay. The three biological replicates were analyzed in triplicate. The error bars indicate the standard deviation; ^#^ *P* < 0.05 and ^###^ *P* < 0.001 (vector Dox- vs. vector Dox+); **P < 0.01 and ***P < 0.001 (Tlk1 Dox− vs. Tlk1 Dox+); ^@^ *P* < 0.05 and ^@@@^ *P* < 0.001 (D607A Dox− vs. D607A Dox+). (**B**) The cell cycle profile of mESCs overexpressing Flag-Tlk1 or Flag-Tlk1-D607A under undifferentiated self-renewal conditions. The cell cycle distribution in Tlk1 overexpression cell lines and control cell lines was analyzed by flow cytometry. The mESCs were cultured in the absence or presence of doxycycline for 48 hrs under undifferentiated self-renewal conditions. The cells were stained with propidium iodide (PI), and the DNA contents were evaluated. The FACS data indicated that the ectopic overexpression of Tlk1 induces the accumulation of G_2_/M phase cells in mESCs irrespective of kinase activity. (**C**) Quantification of FACS data from (**B**). Mean ± S.E.M. of the data from the three biological replicates. *Р < 0.05, **Р < 0.01, and ***Р < 0.001. (**D**) Western blots of whole cell lysates from mESCs expressing Flag-tagged Tlk1 or Flag-Tlk1-D607A under the control of doxycycline were probed with the designated antibodies. The cells were maintained under undifferentiated self-renewal conditions and treated with or without doxycycline for 24 or 48 hrs.

To assess if the ectopic overexpression of Tlk1 induces growth defects in mESCs, we examined the growth rates using a CCK-8 assay. The mESCs stably expressing empty vector or either the Tet-On-Tlk1 or the Tet-On-Tlk1-D607A expression vector were cultured with or without doxycycline for specified times under undifferentiated self-renewal conditions. The growth rate of each cell line was evaluated using a CCK-8 assay. Our results indicated that the overexpression of Flag-tagged Tlk1 or Flag-Tlk1-D607A abolished the ability of mESCs to proliferate, suggesting that the precise control of Tlk1 expression is critical for mESC survival irrespective of the kinase activity of Tlk1 (Fig. 6A). To elucidate the growth defects in Tlk1-overexpressing cells, we investigated cell cycle progression using fluorescence-activated cell sorting (FACS) analysis. Our FACS data revealed that the proportion of wild-type Tlk1 or D607A overexpressing cells in the G_2_/M phase was significantly increased compared to the control mESCs (empty vector or without doxycycline), whereas the proportion of cells in the G_1_- and S-phases was decreased (Fig. 6B and C). Notably, the changes in the cell cycle profile induced by the overexpression of Tlk1 was not observed in the mESCs cultured in the presence of doxycycline for 24 hrs (Supplementary Fig. S3). The data suggest that the defect in cell cycle progression in Tlk1-overexpressing cells could require the extended induction of Tlk1 expression for an additional 24 hrs. Thus, our data suggest that Tlk1 might play a role in cell cycle control in mESCs.

To support the accumulation of cells in the G_2_/M phase induced by the overexpression of Tlk1, we performed an immunoblotting analysis using antibodies against several cell-cycle regulators. We observed that the Wee1 levels and the phosphorylation of CDK1-Tyr15 were decreased in Tlk1 and Tlk1-D607A-overexpressing cells (Fig. 6D). These results were consistent with the observation that the Wee1-mediated phosphorylation of CDK1-Tyr15 is crucial for preventing the premature activation of CDK1 during interphase^27,28^. Moreover, our data indicated that histone H3-Ser10 phosphorylation, a hallmark of M-phase, was increased in Tlk1-overexpressing cells compared to control cells not treated with doxycycline (Fig. 6D). The H3-Ser10 phosphorylation levels in wild-type Tlk1-overexpressing cells were comparable to that of Tlk1-D607A overexpressing mESCs, suggesting that the increased phosphorylation of histone H3-Ser10 in Tlk1- or Tlk1-D607A-overexpressing mESCs might not be a direct consequence of its own Tlk1 kinase activity. Therefore, these data suggest that Tlk1 overexpression might induce the premature activation of CDK1 by the downregulation of Wee1 and the subsequent increase in the phosphorylation of histone H3-Ser10, causing a defect in cell-cycle progression in mESCs. Together, our data suggest that the precise and delicate control of Tlk1 expression levels is very important for proper cell-cycle progression, which likely contributes to the proper maintenance of mESC functions.

### Forced expression of Tlk1 induces apoptosis in mESCs

**Figure 7:**
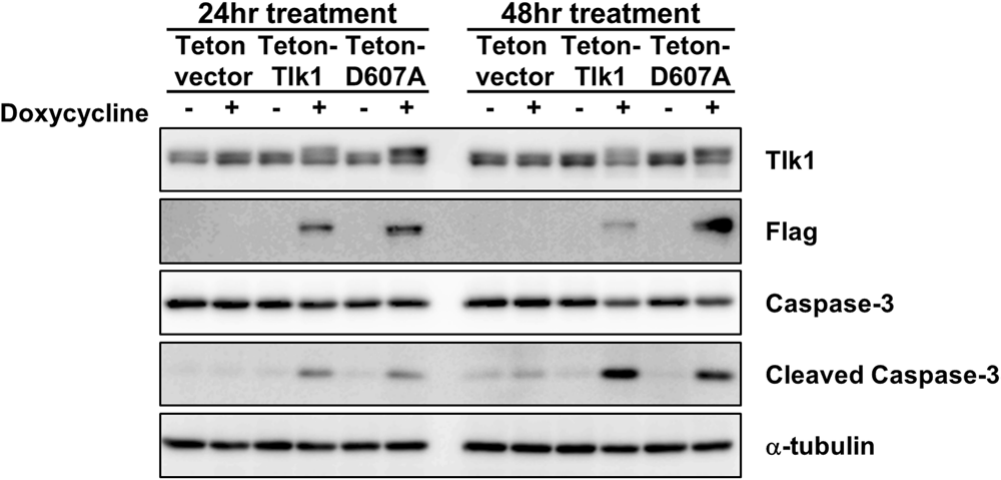
Tlk1 overexpression induces the activation of caspase-3 in mESCs. Western blotting analysis of caspase-3 and activated caspase-3 (cleaved caspase-3) expression was conducted using cells expressing Flag-tagged Tlk1 or the Tlk1-D607A mutant in the absence or presence of doxycycline for 24 or 48 hrs. Alpha-tubulin was used as the loading control.

Because our data demonstrated that Tlk1 overexpression caused an impairment in mESC proliferation, we next attempted to assess if growth defects in Tlk1-overexpressing mESCs might be correlated with the induction of cell death. Hence, we performed an immunoblotting analysis to detect the activation of an apoptotic marker in Tlk1-overexpressing cells. Our data showed that cleaved caspase-3, a hallmark of apoptosis, was clearly detectable 24 and 48 hrs after the induction of Tlk1 or D607A (Fig. 7). Thus, our data suggest that defects in cell cycle control and cell proliferation in mESCs overexpressing Tlk1 might cause apoptosis.

## Discussion

In the present study, we aimed to determine the role of Tlk1 in ESC pluripotency and differentiation. The primary function of mESCs was only marginally affected in *Tlk1*-depleted cells, suggesting that Tlk1 is not required for mESC pluripotency and self-renewal in the undifferentiated state. Further, *Tlk1* depletion caused resistance to differentiation cues and abnormal EB formation, suggesting an impairment in scheduled differentiation. As well, *Tlk1* depletion induced the aberrant expression of both differentiation-associated and pluripotency-associated genes during differentiation. In particular, the downregulation of pluripotency factors during differentiation was blocked in *Tlk1*-depleted mESCs, demonstrating the failure of *Tlk1-*KD cells to differentiate. Conversely, the ectopic expression of Tlk1 was sufficient to induce the untimely downregulation of core pluripotency factors irrespective of kinase activity, thereby leading to a partial loss of self-renewal ability even in the undifferentiated state. Interestingly, we noted that Tlk1 overexpression causes growth defects and an increased number of cells in the G_2_/M phase, as well as apoptosis. The abnormal cell cycle profile was correlated with the increased phosphorylation of histone H3-Ser10 and the downregulation of Wee1 and CDK1-Tyr15 phosphorylation. Collectively, our current data suggest that precise and delicate control of Tlk1 expression levels is critical for proper cell-cycle progression and could contribute to scheduled differentiation.

Both the pluripotency and self-renewal abilities of ESCs are maintained by the expression of specific genes including the transcriptional regulatory circuitry controlled by core TFs such as Oct4, Sox2, and Nanog^29^. The regulatory circuitry serves as a master switch in the establishment and maintenance of the pluripotency state via the positive regulation of several undifferentiated cell markers and the silencing of lineage commitment genes^29^. More specifically, the transcriptional inactivation of a large set of lineage-specific markers by core pluripotency factors is critical for the maintenance of pluripotency and self-renewal^2,16^. In contrast, the pluripotency factors are repressed in a rapid and timely manner in response to differentiation cues, thereby leading to the induction of scheduled differentiation^2^. However, the properties of normal mESCs are largely abrogated in *Tlk1*-depleted cells. Our data indicate that the expression of pluripotency factors are comparatively maintained in *Tlk1*-depleted cells relative to those of control cells during differentiation, concomitant with the aberrant expression of developmental genes (Figs 3 and 4). Consistent with their failure to downregulate the expression of undifferentiated cell markers, *Tlk1*-deficient cells formed irregularly shaped EBs and induced resistance to differentiation cues (Fig. 2), indicating a failure of *Tlk1*-deficient mESCs to differentiate in the context of an EB. The failure of *Tlk1*-depleted mESCs to differentiate is very similar to *BRPF2*- and *Mbd3*-deficient mESCs, in which the scaffold protein BRPF2/BRD1 is a key component of histone acetyltransferase complexes and Mbd3 functions in nucleosome remodeling and the histone deacetylation (NuRD) complex, respectively^25,30^. Together, these data suggest that Tlk1 is required for the scheduled differentiation of mESCs but not for the maintenance of pluripotency and self-renewal in the undifferentiated state.

Tlk1, a serine/threonine kinase, plays important roles in chromatin assembly, DNA repair, and cell cycle progression and phosphorylates Asf1, Rad9, Aurora B kinase, and histone H3^26,31–36^. In *C. elegans*, although TLK-1 kinase activity is not required for enhancing the kinase activity of Aurora kinase B (AIR-2), AIR-2 phosphorylates TLK-1 and, in turn, the phosphorylated TLK-1 reinforces AIR-2 kinase activity, suggesting that TLK-1 is a substrate and activator of the Aurora kinase B^33^. Furthermore, both human TLK1 and AURKB are required for the phosphorylation of histone H3^34,37–39^. These studies could indicate that mouse Tlk1 is potentially associated with the highly conserved mouse Aurkb. Very recently, an elegant study demonstrated that the Aurkb/PP1-mediated resetting of Oct4 during the cell cycle is crucial for determining the identity of mESCs^40^. The phosphorylation of Oct4 by Aurora kinase B during the G_2_/M phase caused Oct4 to dissociate from chromatin, whereas PP1-mediated Oct4 dephosphorylation is required for Oct4 to reoccupy chromatin during exit from the M phase^40^. Interestingly, an Oct4 phosphomimetic mutant that mimicked the Aurkb-mediated phosphorylation of Oct4 caused the cells to lose pluripotency^40^. The authors proposed that the Aurkb/PP1-mediated Oct4 phosphorylation/dephosphorylation cycle plays a potential role in the cell-cycle-dependent control of pluripotency and self-renewal. In the present study, we showed that Tlk1 overexpression caused growth defects, the accumulation of cells in the G_2_/M phase, and apoptosis in mESCs (Figs 5–7). As well, the G_2_/M arrest in Tlk1-overexpressing cells was correlated with increased histone H3 H3-Ser10 phosphorylation, a mitotic marker, and with the downregulation of Wee1 and CDK1-Tyr15 phosphorylation (Fig. 6). These results suggest that the Tlk1-mediated control of scheduled differentiation in mESCs might be demonstrated via Tlk1-dependent cell cycle control. Notably, the Oct4 protein levels were noticeably reduced in Tlk1-overexpressing cells (Fig. 5), suggesting a negative role of Tlk1 in Oct4 expression. After 2 hr release from G_2_/M-arrest by nocodazole, Oct4 phosphorylation was slightly increased in Tlk1-overexpressing cells treated with doxycycline compared to control cells without doxycycline (Supplementary Fig. S5). Based on our study and other reports, we hypothesize that mouse Tlk1 is a substrate and activator of Aurora kinase B as in *C. elegans*; thus, the ectopic and persistent expression of Tlk1 might activate Aurkb, possibly leading to enhanced phosphorylation of Oct4 and the subsequent persistent dissociation of Oct4 from the target promoters. Further, this persistent dissociation of Oct4 from chromatin might cause the loss of self-renewal in mESCs. These findings may raise the possibility that Tlk1 may interact with Aurora kinase B (Aurkb) to control mESC function.

The ectopic expression of Tlk1 leads to the inhibition of cell proliferation in mESCs, which is likely due to G_2_/M phase arrest (Fig. 6). The growth defects and G_2_/M arrest in Tlk1-overexpressing mESCs occur irrespective of its kinase activity. However, in the case of human Tlk1, the overexpression of the wild-type Tlk1 resulted in a normal diploid karyotype, whereas a dominant negative Tlk1 mutant (kinase dead) caused chromosome missegregation and aneuploidy^41^. This discrepancy between human and mouse Tlk1 might be due to species-related differences or cell line-specific differences.

In summary, our results indicate that a *Tlk1* deficiency in mESCs disturbs scheduled differentiation. As well, we found that only a moderate increase in the Tlk1 level is sufficient to downregulate the expression of core pluripotency factors, even under undifferentiated self-renewal conditions, which subsequently leads to markedly reduced cell proliferation, an increased number of cells in the G_2_/M phase, and apoptosis. The G_2_/M arrest observed in Tlk1-overexpressing mESCs correlated with the enrichment of histone H3-Ser10 phosphorylation, a mitotic marker, and with a reduction in the Wee1 and CDK1-Tyr15 phosphorylation levels. Accordingly, we propose that the Tlk1-mediated cell cycle control functions prominently in the lineage commitment of mESCs, as Tlk1 acts as a negative regulator of core pluripotency factor expression.

## Methods

### Cell culture and differentiation

All the mESCs in this study were generated from mouse E14 ESC lines and were maintained in a Knockout Dulbecco’s Modified Eagle’s Medium (DMEM; Gibco, Thermo Fisher Scientific, Waltham, MA) supplemented with 15% (v/v) of EquaFETAL (Cat. #EF-0500-A; Atlas Biologicals, Fort Collins, CO), 2 mM L-Glutamine, 1 × MEM Non-essential Amino Acid (NEAA; Welgene Inc., Gyeongsangbuk-do, Republic of Korea), 1 × penicillin-streptomycin solution (Cat. #30-002-cl; Corning, Mediatech Inc., Manassas, VA), 20 μg/mL ciprofloxacin (Sigma-Aldrich), 55 μM 2-mercaptoethanol (Gibco, Thermo Fisher Scientific, Waltham, MA), and 1000 U/mL of leukemia inhibitory factor (LIF; Cat. #GSR-7001; Global Stem, Gaithersburg, MD). ESCs were cultured in 0.1% gelatin-coated dishes and incubated at 37 °C and 5% CO_2_. Tlk1-knockdown stable cell lines were maintained in the same media as mentioned above with the addition of puromycin (2 µg/ml; Cat. #sc-108071B; Santa Cruz Biotechnology, Inc., Dallas, TX). Doxycycline-induced expression cell lines were maintained in the same media supplemented with puromycin (2 µg/ml) and G-418 (600 µg/ml; Cat. #G0175.0001; Duchefa). 293FT cells were grown in DMEM (Cat. #LM 001-05; Welgene Inc., Gyeongsangbuk-do, Republic of Korea) supplemented with 10% (v/v) fetal bovine serum (FBS) and 1 × penicillin-streptomycin solution (Cat. #30-002-cl; Corning, Mediatech Inc., Manassas, VA) at 37 °C in a humidified atmosphere of 5% CO_2_. For G_2_/M arrest, Tet-On-Tlk1 stable cell lines were treated with 200 ng/ml nocodazole for 10 hr and for release, the cells were washed in PBS for three time. To induce ectopic Tlk1 expression, 100 ng/ml doxycycline was treated to the cells treated with nocodazole for 6 hr until indicated amount of time. To differentiate ES cells, cells were cultured in the medium without LIF (LIF withdrawal, EB and RA). To form embryoid body (EB), modified hanging drop method from previously described protocol^42^ was performed. The cells were plated at a density of 4000 cells per 30 µl drop (20% FBS) in the lids of petri dishes for 2 days. And then hanging drop EBs were transferred to petri dish (“day 0”) and incubated on an orbital shaker at 40 rpm for 3 days (“day 3”) in 5% CO_2_ at 37 °C. Both sphericity and volume of day 3 EBs were analyzed by AnaSP software^43^. All-trans retinoic acid (RA; Cat. #R2625-100 mg; Sigma-Aldrich) was added for 72 hrs before being harvested^44^.

### Lentiviral production and infection

shRNAs targeting Tlk1 was cloned to the pLKO.1-TRC cloning vector (Addgene plasmid 10879; kindly provided by Dr. David Root). shTlk1#1 targets the 3′untranslated region and shTlk1#2 targets the coding sequence region. To generate lentiviruses expressing shRNAs targeting Tlk1, 293FT were transfected with 3 µg of the pLKO.1 shRNA vector, 2.25 µg of pMD2.G (Addgene plasmid 12259; kindly provided by Dr. Didier Trono) and 6.75 µg of ps.PAX2 (Addgene plasmid 12260; kindly provided by Dr. Didier Trono) using Lipofectamine 2000 transfection reagent (Cat. #11668019; Invitrogen)^45^. The medium was replaced with the fresh culture medium 24 hrs after transfection. After cultivating at 37 °C for 24 hrs, the medium was collected and centrifuged at 3000 rpm for 15 min at 4 °C. And the viral supernatant was collected and used to infect ES cells. To knockdown Tlk1 in ES cells, E14 ESCs were infected with pLKO.1 shTlk1 viral particles with 6 µg/ml polybrene for overnight and the medium was replaced by fresh medium. After 24 hrs, the infected cells were selected with 2 µg/ml puromycin.

### Alkaline Phosphatase (AP) staining

AP staining was performed following the manufacturer’s instructions (Cat. #SCR004; Merck Millipore). Briefly, cells were cultured for 5 days, fixed with 4% paraformaldehyde, washed in a rinse buffer and stained with Alkaline Phosphatase Detection Kit.

### RNA Isolation and Quantitative RT-PCR

Total RNA was isolated with TRI Reagent (Cat. #TR118; Molecular Research Center Inc., Cincinnati, OH) and transcribed into cDNA using RevertAid First Strand cDNA Synthesis Kit (Cat. #K1622; Thermo Scientific). qPCR was performed using the Applied Biosystems 7300 Real-Time PCR system and the SYBR® Premix Ex™ Taq II (Tli RNaseH Plus), ROX Plus (Cat. #RR82LRB; Takara, Japan) according to the manufacturer’s instructions.

### Western Blot

Western blot analysis was done as described^46^. Primary antibodies were Oct4 (Cat. #sc-5279; Santa Cruz Biotechnology, Inc., Dallas, TX; 1:2000), Sox2 (Cat. #2748; Cell signaling; 1:1000), Nanog (Cat. #NB100-58842; Novus Biologicals; 1:5000), Tlk1 (Cat. #4125; Cell signaling; 1:5000), alpha tubulin (Cat. #LF-PA0146; Ab Frontier; 1:10000), Flag (Cat. #F3165; Sigma-Aldrich; 1:10000) and anti-p-Oct4 (S229) antibody (GenScript; 1:200).

### Flow Cytometry

Cell cycle analysis was performed as previously described^46^. Briefly, cells were harvested and fixed with cold 70% ethanol at -20 °C for about 3 hrs. And then the fixed cells were stained with propidium iodide and subjected to flow cytometry analysis.

### Immunofluorescence

Cells were cultured in 24-well plate coated with 0.1% gelatin. To differentiate ES cells, cells were cultured for 2 days in the medium without LIF. Cells were fixed with 4% paraformaldehyde, permeabilized with 0.2% Triton X-100 in PBS and incubated with blocking buffer (5% BSA in PBS). Primary antibody was added and incubated for overnight. Cells were washed with PBS followed by adding secondary antibody conjugated with Alexa Flour 488 dyes (1:150 dilution) for 2 hrs at dark room and DAPI solution was added. Cells were visualized as previously described^25^. Primary antibodies were Oct4 (Cat. #sc-5279; Santa Cruz Biotechnology, Inc., Dallas, TX; 1:50), Tlk1 (Cat. #4125; Cell signaling; 1:100) and Nanog (Cat. #NB100-58842; Novus Biologicals; 1:300).

### Inducible overexpression

To conditionally overexpress Tlk1, Lenti-X Tet-On 3 G inducible expression system was used following manufacturer’s protocol (Cat#631353; Clontech). Briefly, wild type Tlk1 and D607A mutant were cloned into pLVX-TRE3G-ZsGreen1 vector. To produce virus particle, 293FT cells were transfected with 7 µg of pLVX-TRE3G vector containing Tlk1 or pLVX-Tet3G vector (regulator), 2.25 µg of pMD2.G and 6.75 µg of psPAX2 using Lipofectamine 2000 transfection reagent. Cells were first infected with regulator virus and selected with G-418. And then pLVX-TRE3G-Tlk1 (WT or D607A) virus infected the cells harboring regulator and the cells was selected by G-418 and puromycin. To induce expression of genes, doxycycline (final concentration 100 ng/ml; Cat. #D9891; Sigma-Aldrich) was treated.

### Cell growth rate assay

Cell growth rate analysis were performed with the Cell Counting Kit-8 (CCK-8 assay kit; Dojindo Corporation, Kumamoto, Japan) as previously described^46^. Briefly, 24 hrs prior to experiments, 1200 cells per well were plated onto each well of a 96-well plate (100 µl medium of cell suspension) and from the next day medium in the absence or presence of doxycycline (final concentration 100 ng/ml) was changed for every day. And after adding CCK-8 solution, absorbance was measured four times at an interval of 24 hrs.

### Statistical analysis

Data are presented as the means ± S.E.M. or means ± S.D. Two-tailed student’s t-tests were performed to analyze the data between controls and experimental groups. Statistical significance (*P* value) is indicated for each graph as asterisks (*P < 0.05, **P < 0.01, ***P < 0.001).

## Electronic supplementary material

Supplementary data
