# Supplementary material for: Tousled-like kinase 1 is a negative regulator of core transcription factors in murine embryonic stem cells
Source: Sci Rep. 2018 Jan 10;8:334. doi: 10.1038/s41598-017-18628-9 (PMC5762884; doi:10.1038/s41598-017-18628-9)
Supplement: Supplementary file 1 — Supplementary data [file 41598_2017_18628_MOESM1_ESM.pdf]

Supplementary Data

Tousled-like kinase 1 is a negative regulator of core transcription factors in murine embryonic stem cells

Jina Lee, Min Seong Kim, Su Hyung Park and Yeun Kyu Jang

Inventory of Supplemental Information

The Supplemental Information contains 8 additional figures, and 2 tables

Figure S1: Related to Figures 2 and 3

Figure S2: Related to Figure 5.

Figure S3: Related to Figure 6.

Figure S4: Related to Figure 5.

Figure S5: Related to Figure 5.

Figure S6: Uncropped gel image from Western blot: Fig. 1E, Fig. 4A, Fig. 4C, Fig. 4E, and Fig. 5A

Figure S7: Uncropped gel image from Western blot: Fig. 6D, Fig. 7, Fig. S1D and Fig. S1E.

Figure S8: Uncropped gel image from Western blot: Fig. S4 and Fig. S5.

Table S1: shRNA target sequences

Table S2: Primer sequences for qRT-PCR

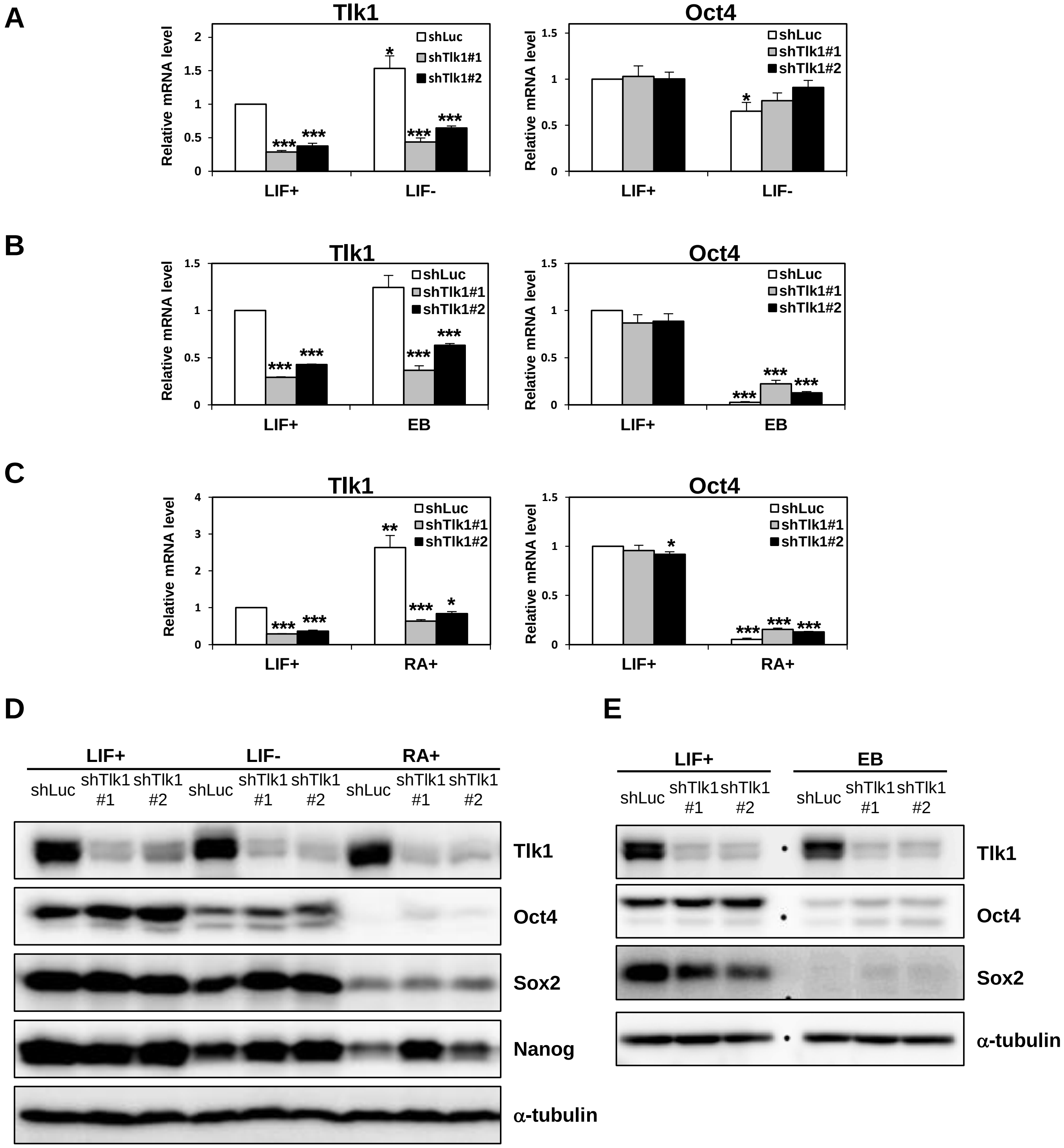

Supplementary Figure S1 (Related to Figures 2 and 3). The *Tlk1*-KD mESCs successfully differentiated. (A-C) The mRNA levels of Tlk1 and Oct4 in *Tlk1*-KD mESCs upon differentiation. All data are normalized to Gapdh. The mRNA levels in luciferase (Luc)-KD control cells in the presence of LIF were normalized to 1. Data are means (n=3) ± SEM for LIF- and EB and for RA (n=4). * =P<0.05, ** = P<0.01, and *** = P<0.001. (D-E) Western blots of Tlk1 and pluripotency markers in Tlk1-KD mESCs.

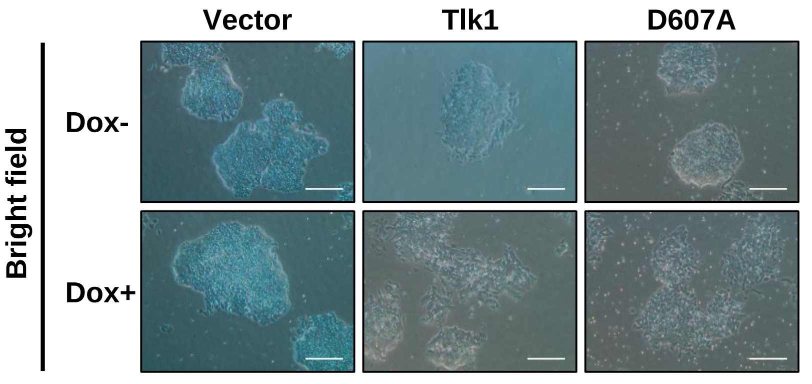

Supplementary Figure S2 (Related to Figure 5). Ectopic Tlk1 expression negatively regulates self-renewal. The morphology of the TetOn-Tlk1 inducible cell lines cultured in the absence (Dox-) or presence (Dox+) of doxycycline for 24 hrs. Symbols: Tlk1, wild-type; D607A, kinase-dead mutant form of Tlk1. Scale bar, 200 µm.

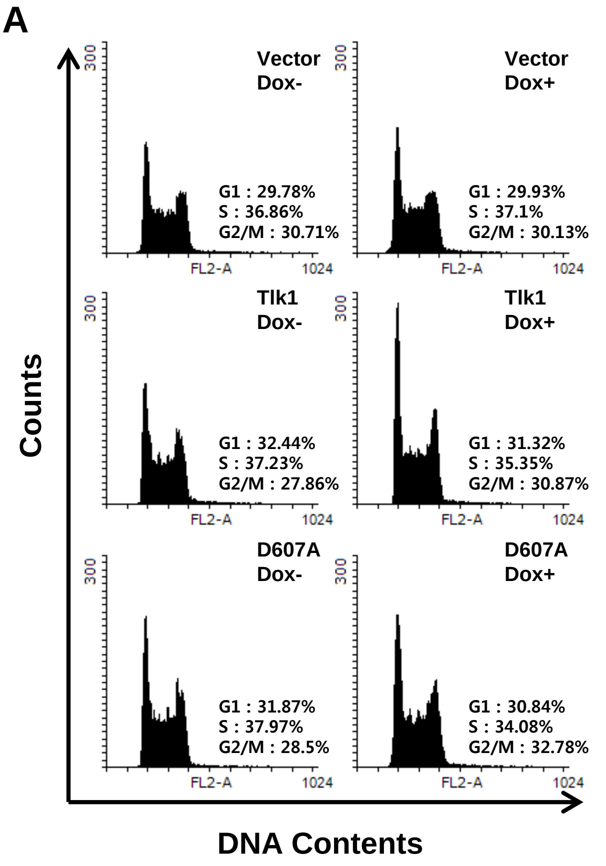

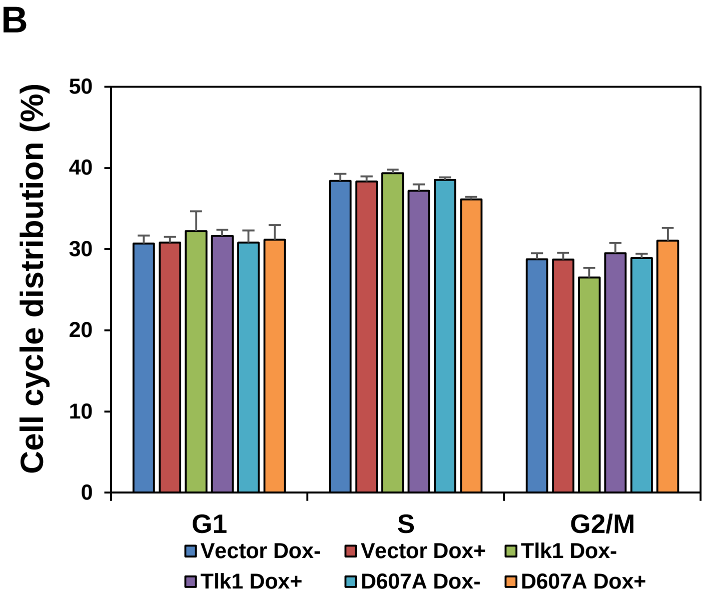

	Control		Tlk1		D607A	
	Dox-	Dox+	Dox-	Dox+	Dox-	Dox+
G1	30.68±1.0	30.81±0.7	32.24±2.4	31.62±0.8	30.82±1.5	31.15±1.8
S	38.40±0.9	38.35±0.6	39.37±0.4	37.17±0.8	38.52±0.3	36.11±0.3
G2/M	28.73±0.8	28.71±0.9	26.49±1.2	29.50±1.3	28.93±0.5	31.04±1.6

Supplementary Figure S3 (Related to Figure 6). The effect of Tlk1 on cell cycle progression 24-hr after induction of Tlk1 by doxycycline treatment. (A) Representative histograms of Teton-Tlk1 inducible cell lines in the absence or presence of doxycycline for 24 hrs. (B) Percentages of cells in G1, S, and G2/M phases. Data are means (n=3) ± SEM.

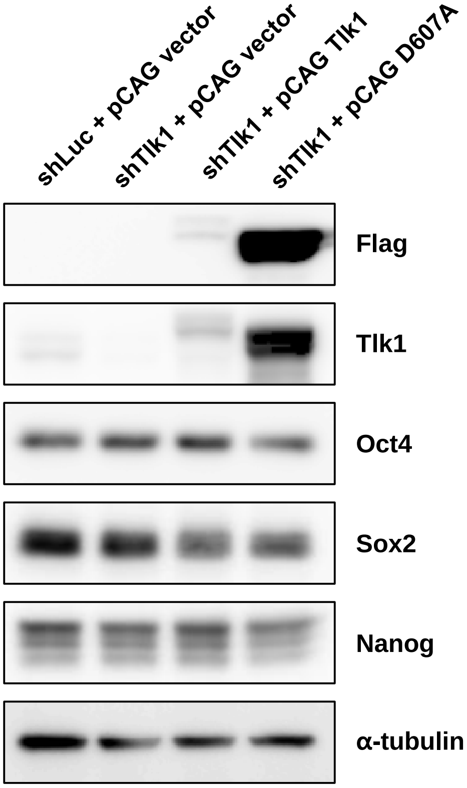

Supplementary Figure S4 (Related to Figure 5). The effect of Tlk1 overexpression on core pluripotency factors in the conditions of depletion of endogenous Tlk1. Western blots of Flag, Tlk1 and pluripotency markers after transient overexpression of Tlk1 in *Tlk1*-KD mESCs.

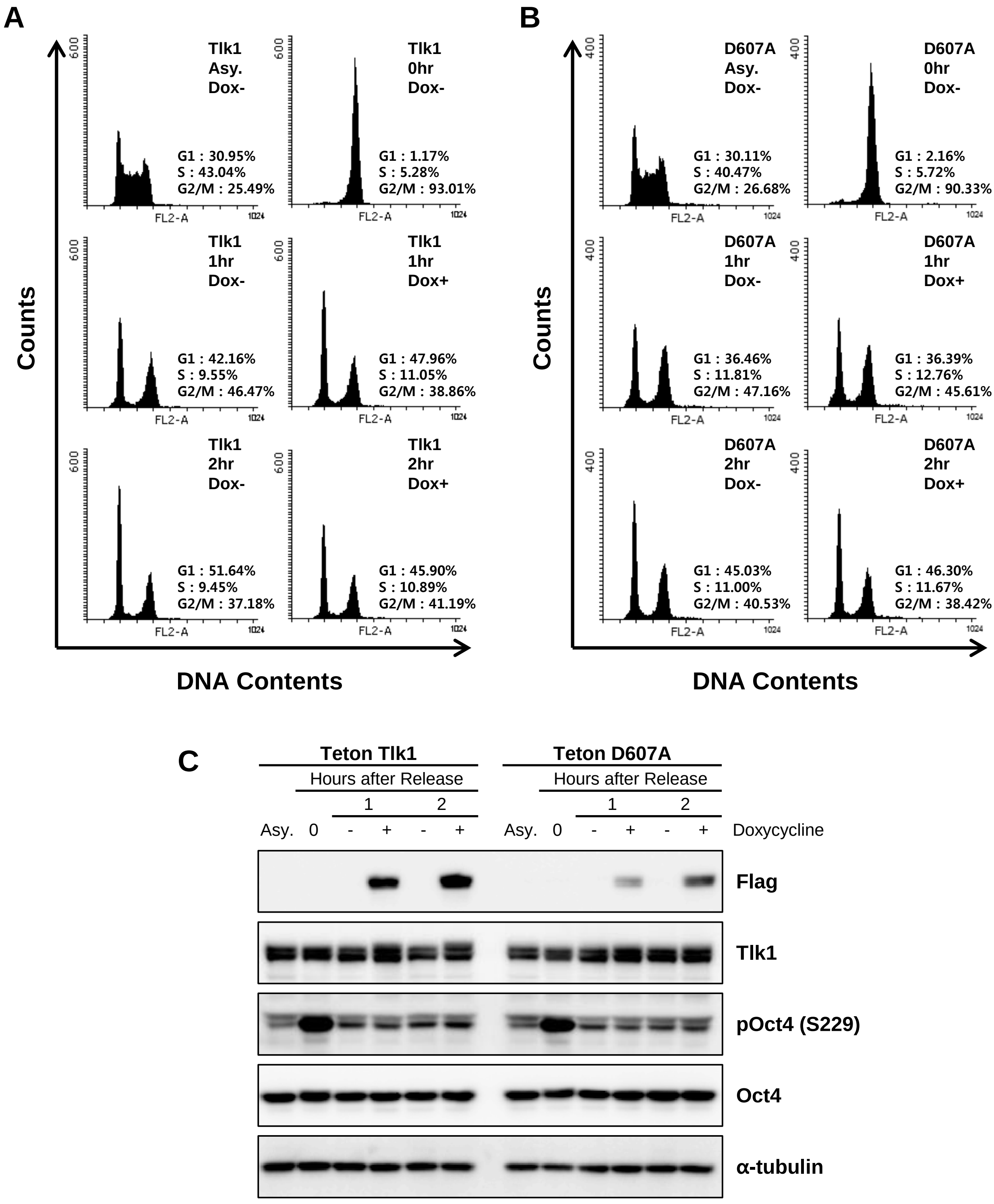

Supplementary Figure S5 (Related to Figure 5). The effect of Tlk1 overexpression on Oct4 phosphorylation (S229) in mESCs. (A, B) Histograms of the Tet-On-Tlk1 or Tet-On-Tlk1-D607A inducible cell lines at different time points after release from a nocodazole arrest. (C) Western blots of whole cell protein extracts from Tet-On-Tlk1 or Tet-On-Tlk1-D607A inducible cell lines released from a nocodazole arrest were probed with the indicated antibodies. Symbols: Asy., asynchronous

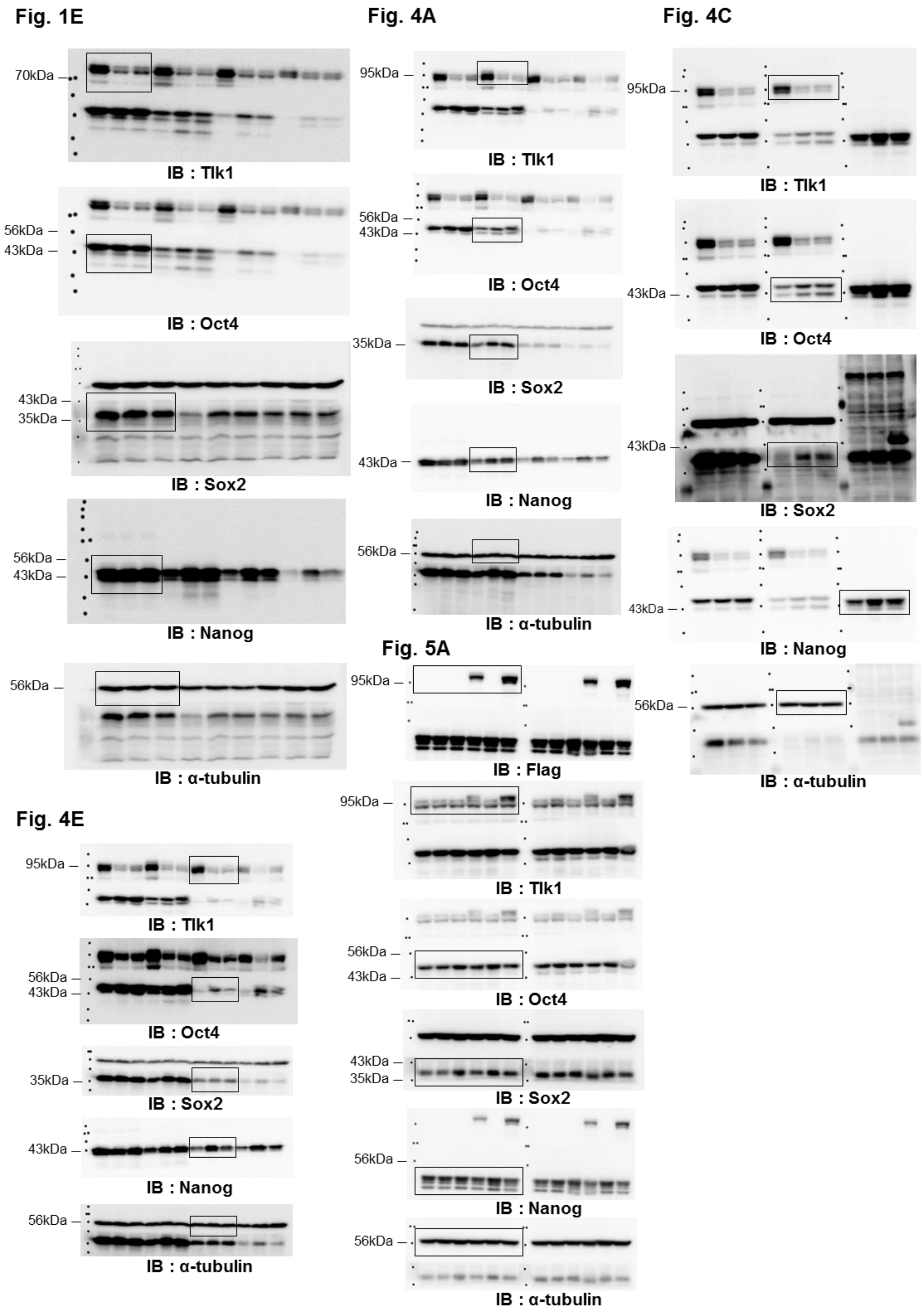

Supplementary Figure S6. Uncropped gel image from Western blot: Fig. 1E, Fig. 4A, Fig. 4C, Fig. 4E, and Fig. 5A

Fig. 6D

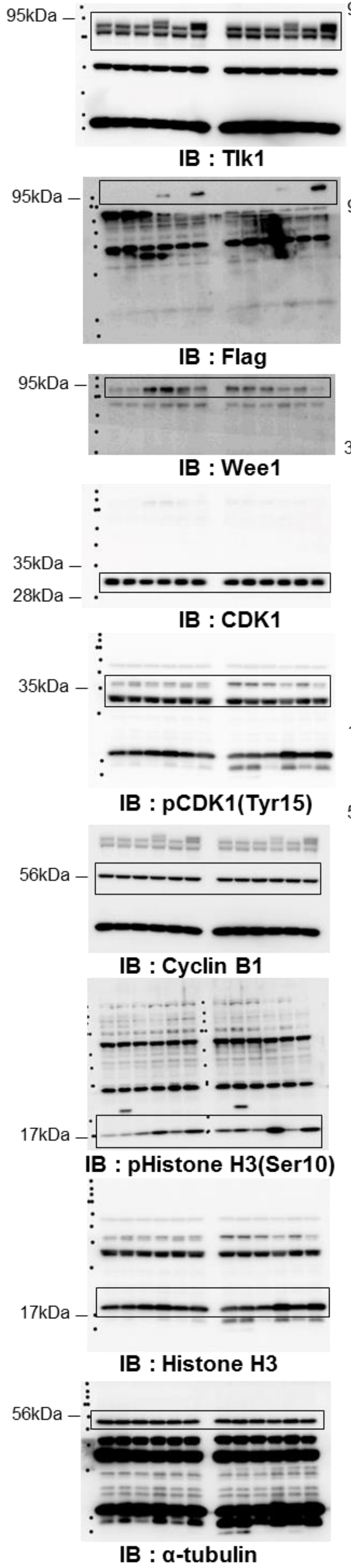

Fig. 7

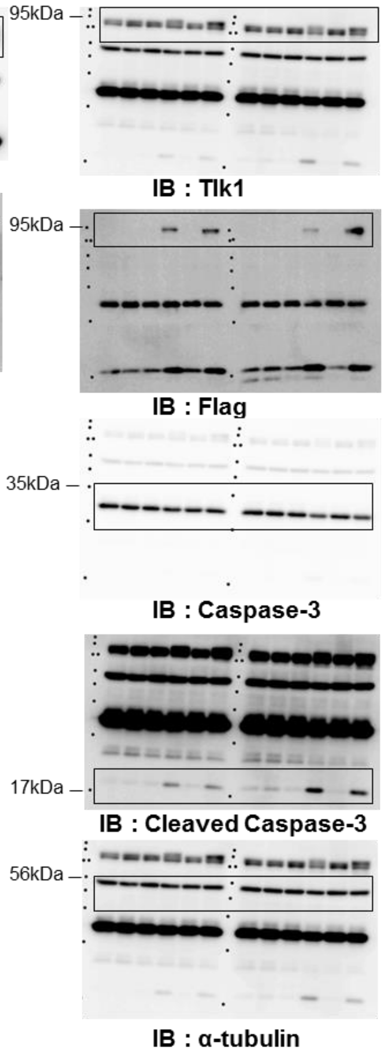

Supplementary Fig. S1D

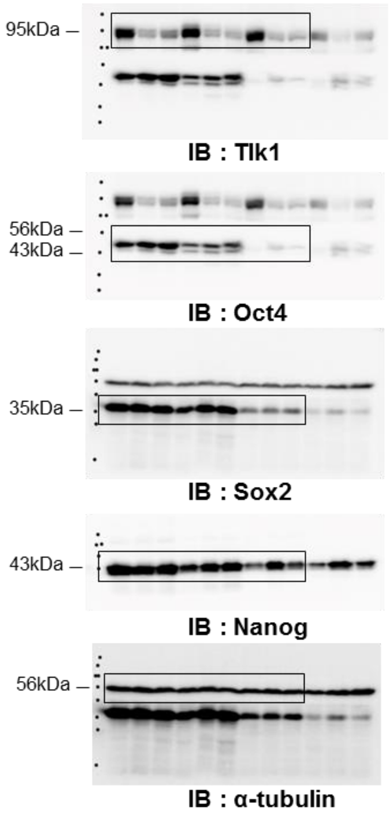

Supplementary Fig. S1E

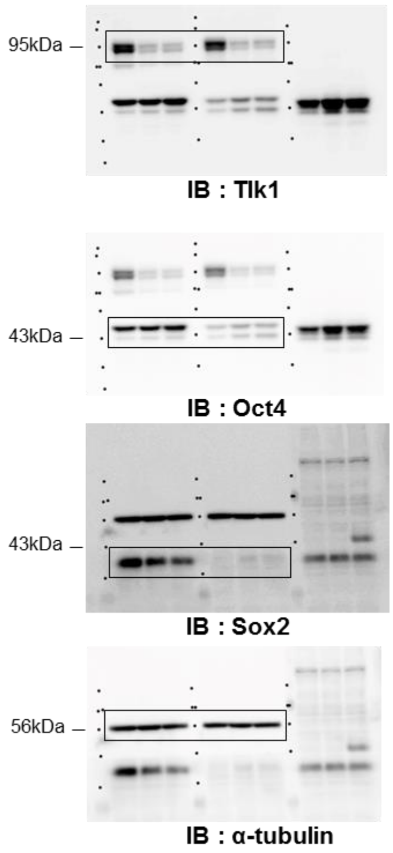

Supplementary Figure S7. Uncropped gel image from Western blot: Fig. 6D, Fig. 7, Fig. S1D, & Fig. S1E

Supplementary Fig. S4

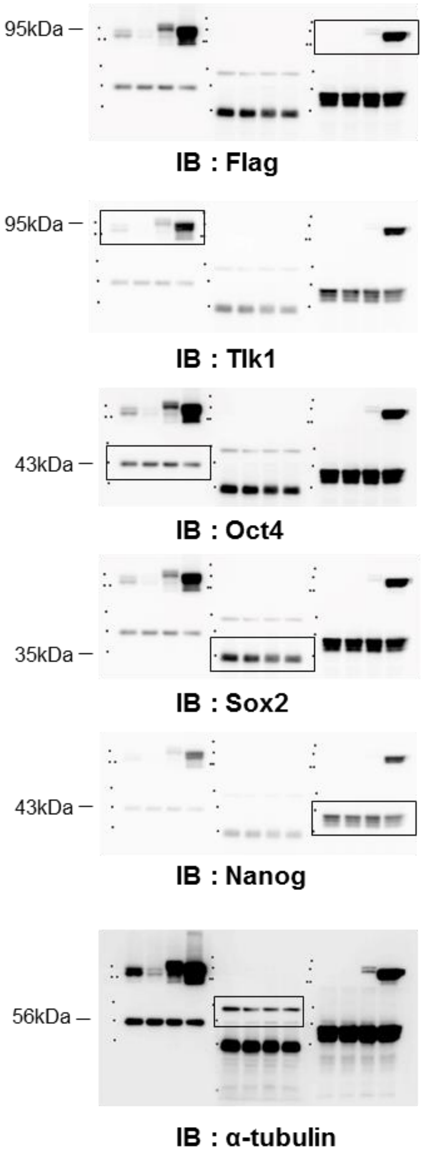

Supplementary Fig. S5

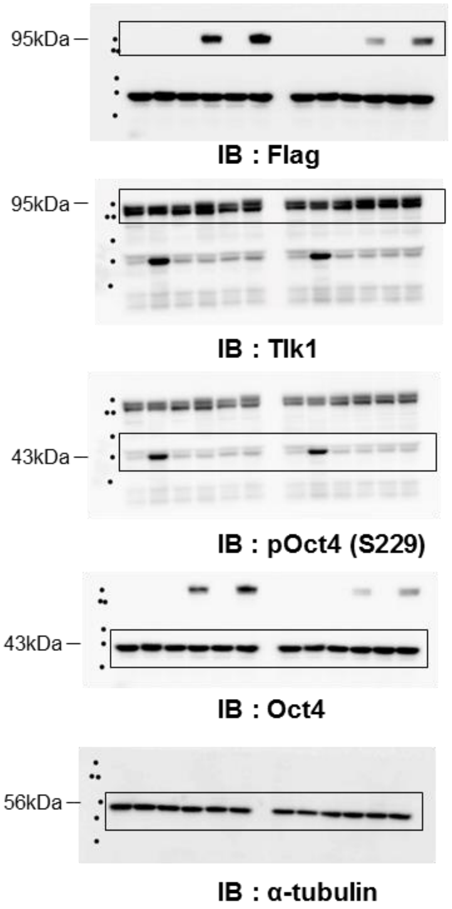

Supplementary Figure S8. Uncropped gel image from Western blot: Fig. S4, & Fig. S5

Supplementary Table S1. shRNA target sequences

Name	Sequences
Luciferase(Luc)	AGAGCTGTTTCTGAGGAGCCT
Tlk1#1(3’UTR)	GCGCTTTAATTGCTGCTAAAT
Tlk1#2(CDS)	CGGCTTTAGTGAGGTCTATAA

Supplementary Table S2. Primer sequences for qRT-PCR

Gene	Forward sequence	Reverse sequence
Tlk1	TGGAACAGCATGTGGAGA	CCTGGGAAGTCAGATCCA
Oct4	AGAAGTGGGTGGAGGAAG	GGCACTTCAGAAACATGG
Sox2	ACAACTCGGAGATCAGCA	TCATGAGCGTCTTGGTTT
Nanog	TGCACTCAAGGACAGGTT	TGCACTTCATCCTTTGGT
Klf2	CCACTACCGAAAGCACAC	AGCAGTCTGTTTGCAAGG
Klf5	CACCTGAGGACTCATACG	GTGTGCTTCCTGTAGTGG
Esrrb	CTACTAGGGGTTGAGCAGGA	CCAGTTGATGAGGAACACAA
Max	GAAAACGTAGGGACCACATC	GTGCGTATGGTTTTTCCTTC
Tfcp2l1	TCCTGGAAGAGCTGACTACC	CATCTCATTGCTTACCACCA
Cdx1	ACGCCCTACGAATGGATG	CTTGGTTCGGGTCTTACCG
Otx2	CATGATGTCTTATCTAAAGCAACCG	GTCGAGCTGTGCCCTAGTA
Flk1	TTCATCGCCTCTGTCAGT	CTGGATACCTAGCGCAAA
Nkx2.5	ATTTTACCCGGGAGCCTA	GCTTTGTCCAGCTCCACT
Gata4	ATCAAACCCCTTGCTCTC	AGGTGCAGATGAGCCATA
Gata6	GCAAGATGAATGGCCTCAGC	AAGCATTGCACACAGGCTCA
Fgf5	ACCGGAACTTTCCTTCAC	CATCATCCAAAGCGAAAC
Tubb3	TTCCCACGTCTCCACTTC	GGCAGCCATCATGTTCTT
Id2	AGAGACCTGGACAGAACC	CGACATAAGCTCAGAAGG
Hand1	AAGGATGCACAAGCAGGT	GAGGCAACTCCCTTTTCC
